# Sex and Age Aspects in Patients Suffering From Out-Of-Hospital Cardiac Arrest: A Retrospective Analysis of 760 Consecutive Patients

**DOI:** 10.1097/MD.0000000000003561

**Published:** 2016-05-06

**Authors:** Tobias Piegeler, Nils Thoeni, Alexander Kaserer, Martin Brueesch, Simon Sulser, Stefan M. Mueller, Burkhardt Seifert, Donat R. Spahn, Kurt Ruetzler

**Affiliations:** From the Institute of Anesthesiology, University and University Hospital Zurich (TP, NT, AK, MB, SS, DRS, KR); Schutz und Rettung, Ambulance Service, Zurich, Switzerland (SMM); Epidemiology, Biostatistics and Prevention Institute, Department of Biostatistics, University of Zurich, Switzerland (BS); and Department of Outcomes Research and General Anesthesiology, Anesthesiology Institute, Cleveland Clinic, Cleveland, OH (KR).

## Abstract

Cardiopulmonary resuscitation (CPR) is indicated in patients suffering from out-of-hospital cardiac arrest. Several studies suggest a sex- and age-based bias in the treatment of these patients. This particular bias may have a significant impact on the patient's outcome. However, the reasons for these findings are still unclear and discussed controversially. Therefore, the aim of this study was to retrospectively analyze treatment and out-of-hospital survival rates for potential sex- and age-based differences in patients requiring out-of-hospital CPR provided by an emergency physician in the city of Zurich, Switzerland.

A total of 3961 consecutive patients (2003–2009) were included in this retrospective analysis to determine the frequency of out-of-hospital CPR and prehospital survival rate, and to identify potential sex- and age-based differences regarding survival and treatment of the patients.

Seven hundred fifty-seven patients required CPR during the study period. Seventeen patients had to be excluded because of incomplete or inconclusive documentation, resulting in 743 patients (511 males, 229 females) undergoing further statistical analysis. Female patients were significantly older, compared with male patients (68 ± 18 [mean ± SD] vs 64 ± 18 years, *P* = .012). Men were resuscitated slightly more often than women (86.4% vs 82.1%). Overall out-of-hospital mortality rate was found to be 81.2% (492/632 patients) with no differences between sexes (82.1% for males vs 79% for females, odds ratio 1.039, 95% confidence interval 0.961–1.123). No sex differences were detected in out-of-hospital treatment, as assessed by the different medications administered, initial prehospital Glasgow Coma Scale, and prehospital suspected leading diagnosis.

The data of our study demonstrate that there was no sex-based bias in treating patients requiring CPR in the prehospital setting in our physician-led emergency ambulance service.

## INTRODUCTION

Cardiopulmonary resuscitation (CPR) is indicated in patients suffering from out-of-hospital cardiac arrest. Early access to professional medical treatment—including CPR, defibrillation, and advanced cardiac life support—as well as a high standard of care after resuscitation are crucial in out-of-hospital emergency care.^1^

The overall survival rate on admission after out-of-hospital CPR has previously been reported to be about 12%, whereas survival to hospital discharge ranged between 2% and 7.6%.^1–3^ The patients’ outcome after cardiac arrest is at least in part affected by the quality of the CPR provided.^4^ Several studies have previously reported that the CPR quality might be affected by age,^4,5^ the presence of bystander CPR,^6^ early defibrillation, and even the patient's sex.^6,7^ Several studies also revealed the fact that women are more likely to survive out-of-hospital cardiac arrest (especially due to ventricular fibrillation) and CPR.^6–8^ However, the reasons for these interesting findings are still unknown and currently discussed extensively.^9^

Therefore, the aim of this retrospective analysis was to evaluate potential sex- and age-related differences regarding survival and treatment in patients suffering from out-of-hospital cardiac arrest who were treated by an emergency physician in the city of Zurich, Switzerland.^10^ To our knowledge, this is the first clinical report focusing on potential sex aspects in patients undergoing out-of-hospital CPR in Switzerland.

## METHODS

### Data Collection

In accordance with the guidelines of local ambulance service “Schutz und Rettung Zurich (SRZ),” personal and medical data for each patient were recorded on a paper-based report form, stored, and archived after the end of the case. With approval of the local ethics committee (Kantonale Ethikkommission Zurich, Switzerland, KEK-ZH-Nr. 2014–0372), we screened these report forms for all out-of-hospital emergency patients (older than 18 years) requiring CPR (National Advisory Committee for Aeronautics –NACA- score of 6) or pronounced dead (NACA score of 7) in the city of Zurich, Switzerland, between 2003 and 2009.

Prehospital survival was defined as a sustained return of spontaneous circulation until admission to the hospital (a “survived event” in accordance with the Utstein guidelines).^11^

Additionally, we separated these patients into 2 groups:

1. Patients undergoing cardiopulmonary resuscitation (CPR group) and
2. Patients pronounced dead or not undergoing out-of-hospital CPR

Possible sex aspects were also assessed in these subgroups.

### Statistical Analysis

Age is presented as mean with standard deviation (SD) and compared between sexes using a Student *t* test for independent samples. Qualitative data are expressed as the number of patients and the corresponding percentage of the total group. Proportions between groups were compared using the *χ*^2^ and Fisher exact test, where appropriate. Odds ratios (ORs) are presented with corresponding 95% confidence intervals (95% CIs). After correcting for the sex of the patients, multivariable logistic regression was used to evaluate age-related effects on out-of-hospital survival rate.

A *P* value of <0.05 was considered to be statistically significant. All data were analyzed using IBM SPSS Statistics (Version 22.0, IBM Corp., Armonk, NY).

## RESULTS

A total of 3961 patients were treated by an emergency physician in the out-of-hospital setting during the study period. Of these, 757 patients had a NACA score of 6 or 7. Incomplete or inconclusive documentation lead to an exclusion of 17 patients. The remaining 740 patients (511 males, 229 females) underwent further analysis.

Overall out-of-hospital survival rate of all patients suffering from an out-of-hospital cardiac arrest—whether resuscitated or not—was found to be 18.9 % (140/740 patients) with no differences between sexes (18% for males vs 21% for females, OR 0.859, 95% CI 0.629–1.174).

Female patients were significantly older than the male patients (67.6 ± 17.9 [mean ± SD] vs 63.9 ± 18.2 years, *P* = .012). Six hundred thirty-one of the 740 patients (85.3 %) underwent out-of-hospital CPR. There were no significant differences in out-of-hospital survival rate between the 2 sexes of resuscitated patients: resuscitated men reached the hospital alive in 20.8% and women in 25.5% of the cases (OR 0.813, 95% CI 0.600–1.103). Men were resuscitated slightly more often than women (86.7% vs 82.1%), although this difference was not statistically significant (OR 1.056, 95% CI 0.985–1.132).

To assess the effect of patient age on out-of-hospital survival, patients were grouped into quintiles (n = 148 each). Patients had a mean age of 37 years in quintile 1 (18–50, minimum–maximum), a mean age of 57 years (51–63) in quintile 2, and a mean age of 68 years (63–73) in quintile 3. The patients in quintiles 4 and 5 had mean ages of 78 (73–82) and 86 (82–97) years, respectively.

In a logistic regression, age analyzed in quintiles was a significant risk factor for out-of-hospital mortality (*P* = .013). Overall out-of-hospital survival was higher in quintile 2 (mean age of 57 years) compared with the reference category (quintile 1, mean age 37 years) and then decreased with age. The result remained unchanged after adjusting for the sex of the patients. Overall out-of-hospital survival remained similar between sexes after adjustment for age (OR 1.24, 95% CI 0.83–1.83).

There were no significant differences between the quintiles regarding the overall rate of attempted CPR (Fisher exact test, *P* = .18). However, patients in quintile 2 (mean age of 56.7 years) showed a significantly higher out-of-hospital survival rate compared with the other groups (33.9% vs 17.1%–22.1%, OR 0.402, 95% CI 0.221–0.732).

Additionally, no sex differences in out-of-hospital treatment, as assessed by the different medications administered (Table 1), were observed. Initial out-of-hospital Glasgow Coma Scale (GCS, Table 2) and out-of-hospital suspected leading diagnosis (Table 3) were also distributed similarly between sexes.

**TABLE 1:**
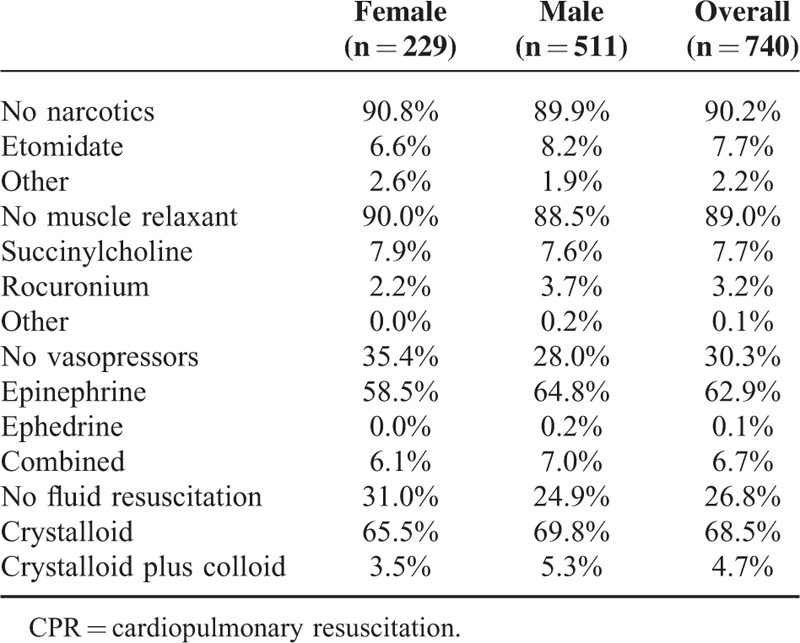
Summary of Out-Of-Hospital Medical Treatment Applied by the Emergency Physician to Either Females or Males Receiving CPR

**TABLE 2:**
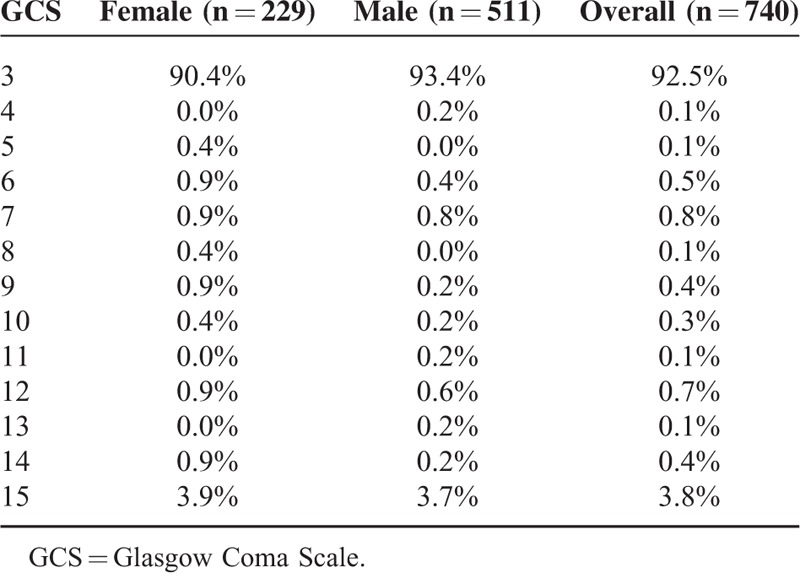
Initial GCS of Patients in Need of Out-Of-Hospital Cardiopulmonary Resuscitation Upon Arrival of the Emergency Physician

**TABLE 3:**
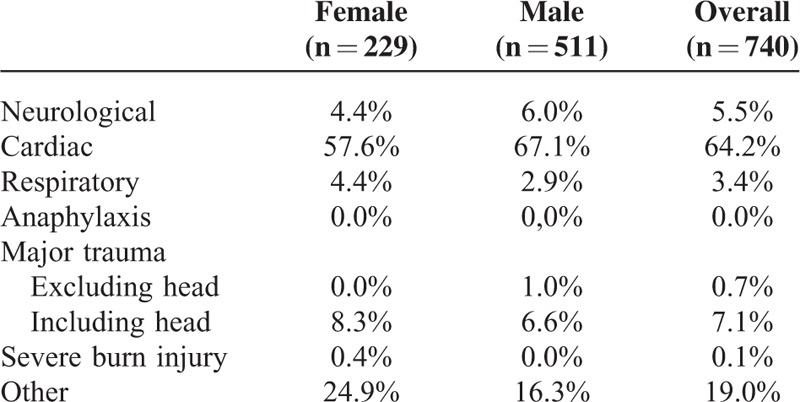
Leading Diagnosis (Assigned by the Emergency Physician) of Patients Receiving Out-Of-Hospital Cardiopulmonary Resuscitation

## DISCUSSION

No sex-based bias in the treatment of patients requiring CPR could be detected in our retrospective analysis. Furthermore, treatment with medications, initial GCS, and the suspected leading diagnosis were distributed similarly between females and males in the out-of-hospital setting.

Previous studies examining differences in patients undergoing out-of-hospital CPR have mainly focused on interactions regarding patient age, the incidence of ventricular fibrillation, and the survival rate after hospital admission, and reported that women are more likely to survive these events than men.^6–8^ Furthermore, the patient's sex has been demonstrated to potentially act as an independent predictor for the outcome. Several studies showed that females—when compared with males—might have a worse outcome because of higher age, less bystander CPR, fewer witnessed events, fewer events in a public location, and less shockable rhythms.^1,6,7,12,13^ Additionally, the finding that female patients tend to be older is supported by the results of our study.

Many studies examining sex and survival rate on admission showed a potential benefit in women.^3^ This could at least in part be confirmed by the data of our study, demonstrating a survival rate on admission of 21% for females and 18% for males, although this difference was not statistically significant. Ahn et al^1^ reported a higher rate of survival on admission for women, when compared with males. Interestingly, the authors also reported similar survival rates until discharge for women and men.^1^ However, data on survival to hospital discharge are conflicting.^3^ The majority of studies support the hypothesis that there might be no sex differences regarding overall survival after out-of-hospital CPR, whereas some studies suggest advantages for women or even the opposite (ie, for men).^3^ Although the discussion about the potential of better survival of women or men after admission is still controversial, the findings of Wachelder et al^14^ also suggest that female survivors might be suffering from a poorer quality of life after out-of-hospital CPR.

The overall out-of-hospital survival rate in our study (18% for males vs 21% for females) did not differ significantly between women and men, but was comparable with the findings of other authors.^15^ Akahane et al^16^ previously reported that men might be statistically more likely to survive after an out-of-hospital cardiac arrest even after adjusting for age, which is contrary to the results of the present study.

Previous studies reported an impact of sex-related health disparities on outcome after acute myocardial infarction or stroke. This phenomenon was explained by potential differences in the effects of hormones and socioeconomic status between sexes.^17–20^ However, these studies were all limited to patients suffering from acute myocardial infarction or acute stroke and are thus not completely comparable with the findings of the present study.

It has been demonstrated that age and sex may affect both the decision to perform and the duration of CPR. However, these particular studies were limited by a rather small sample size as well as their patient collective (eg, patients undergoing in-hospital CPR).^21,22^ Kramer et al^9^ recently reported results from a manikin study, showing a potential effect of the sex of the manikin on the quality of CPR: participants were more reluctant to remove clothing from the female manikins and rescuers were more likely to place the hands across the chest toward the nipples during CPR. Although the removal of clothes and hand placement were not investigated, the data of our study might as well confirm these findings, as men were slightly more often resuscitated than women (86.7% vs 82.1%). However, this difference was not statistically significant. Interestingly, another study examining 681 patients from an urban population undergoing out-of-hospital CPR (comparable to our own study setting) showed a higher resuscitation rate for women.^23^

Although mortality did not differ significantly between sexes, the results of our study suggest that mortality was highest in patients aged between 50.8 and 62.8 years. Our data therefore confirm, at least in part, the previous findings by Safdar et al^3^, wherein the authors described that the probability of survival differed across age for men and women in a nonlinear fashion.

### Limitations

Our study has several limitations. First, it is a retrospective analysis and documentation was sometimes incomplete and could therefore not be evaluated. However, the overall quality of data and its documentation were good and only 17 patients had to be excluded because of missing data or incomplete documentation.

Second, initial cardiac rhythms were not documented on a routine basis and could therefore not be analyzed further for the present study.

Third, the results of our study are of course limited to the local circumstances of the city and greater area of Zurich, and may not be applicable to all out-of-hospital emergency services.

In conclusion, the data of our study do not support the idea that there is a sex-based bias in the treatment of patients requiring CPR in the out-of-hospital setting. Female patients requiring CPR in our study were about 4 years older compared with males, but overall out-of-hospital survival rate was comparable between both sexes.
